# Macrophage migration inhibitory factor -173 G > C polymorphism and risk of tuberculosis: A meta-analysis

**DOI:** 10.17179/excli2016-662

**Published:** 2017-03-21

**Authors:** Mohammad Naderi, Mohammad Hashemi, Hossein Ansari

**Affiliations:** 1 Infectious Diseases and Tropical Medicine Research Center, Zahedan University of Medical Sciences, Zahedan, Iran; 2 Department of Clinical Biochemistry, School of Medicine, Zahedan University of Medical Sciences, Zahedan, Iran; 3 Department of Epidemiology and Biostatistics, Zahedan University of Medical Sciences, Zahedan, Iran

**Keywords:** MIF, polymorphism, tuberculosis, meta-analysis

## Abstract

The aim of the present meta-analysis was to find out the impact of *MIF* -173 G > C polymorphism on risk of tuberculosis (TB). We conducted a search of case-control studies on the associations of -173 G > C variant of *MIF* with susceptibility to tuberculosis in PubMed, ISI Web of Science, and Scopus. We extracted the data from eligible studies and achieved a meta-analysis to examine the relationship between *MIF* -173 G > C polymorphism and the risk of TB. Odds ratios (ORs) with the corresponding 95 % confidence intervals (CIs) were pooled to find out the impact of *MIF* -173G > C promoter polymorphism on TB risk. The pooled ORs were calculated for the codominant, dominant, recessive, and allelic model comparison. The findings revealed that *MIF* -173 G > C variant increased the risk of TB in codominant (OR = 1.54, 95 %CI = 1.26-1.88, p < 0.0001; CG vs GG), and dominant (OR = 1.62, 95 %CI = 1.33-1.96, p < 0.00001; GC+CC vs GG) inheritance models tested. The results suggested that the *MIF* -173 C allele significantly increased the risk of PTB (OR = 1.49, 95 %CI = 1.28-1.74, p < 0.00001). The findings of this meta-analysis propose that *MIF* -173 G > C variant is associated with the risk of TB. More case-control studies with well-designed in different ethnic groups and larger sample size are needed to confirm the findings.

## Introduction

Tuberculosis (TB), mainly caused by *Mycobacterium* tuberculosis (*M.* tuberculosis), is a main global public health problem and it is responsible for high mortality and morbidity worldwide particularly in Asia and Africa (Zaman, 2010[33]). World Health Organization (WHO) report proposed that nearly 9 million subjects developed TB in 2014 and 1.5 million people died from the disease (Zumla et al., 2015[35]). Though one third of the world's population has infected latent TB, merely 5-10 % of infected cases will develop the clinical disease (Philips and Ernst, 2012[22]). 

Previous studies in animals and humans have shown that, apart from environmental factors, genetic background of the host may play an important role in the development of TB (Bellamy, 2003[3]; Schurr, 2007[27]; Azad et al., 2012[1]; Meilang et al., 2012[19]; Tong et al., 2015[28]). Until now, several gene polymorphisms have been suggested as being associated with susceptibility to TB (Hashemi et al., 2013[11]; Naderi et al., 2014[20][21]; Yang et al., 2016[32]). 

Human macrophage migration inhibitory factor (MIF) gene is located on long arm of chromosome 22 (22q11.2). The gene consists of three exons of 205, 173 and 183 bp separated by two introns of 189 and 95 bp (Budarf et al., 1997[4]). A -173 G > C functional variant in the promoter region of MIF seems to affect promoter activity in a cell-type dependent manner (Donn et al., 2002[7]; Renner et al., 2005[23]). MIF protein is a multifunctional cytokine that is made by many types of cells such as epithelial cells as well as cells contribute to the innate and adaptive immune responses (Calandra et al., 1994[6]; Bacher et al., 1997[2]). MIF is known as an immunomodulatory cytokine that involved in the initiation of innate immune response by inducing tumor necrosis factor-α (TNF-α), Interferon-γ (IFN-γ), interleukin-2 (IL-2), and IL-6 production during microbial infection (Calandra et al., 1995[5]; Roggero et al., 2002[24]; 2004[25]; Marinho et al., 2007[18]). 

Several studies investigated the impact of *MIF* -173 G > C variant on risk of tuberculosis (Gomez et al., 2007[9]; Sadki et al., 2010[26]; Li et al., 2012[15]; Hashemi et al., 2013[11]; Kuai et al., 2016[14]; Liu et al., 2016[16]). The present study aimed to perform a meta-analysis of all eligible studies to evaluate the overall association between the *MIF *-173 G > C polymorphism and risk of tuberculosis. 

## Methods

**Figure 1:**
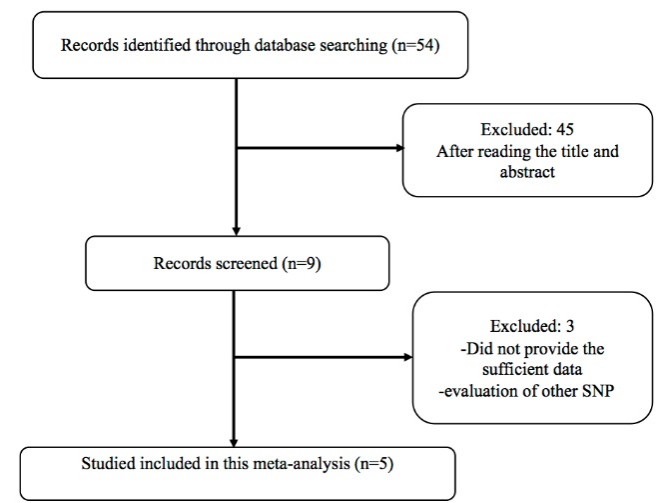
Flow chart of literature screening and selection in the meta-analysis

A comprehensive search of PubMed, Web of Science, Scopus, and Google Scholar was done from database for articles published up to April 04, 2016 without language restriction. The search strategy was “macrophage migration inhibitory factor or MIF” and “polymorphism or variant or mutation or genotype” and tuberculosis. Relevant studies which were eligible for the meta-analysis must meet the following criteria: studies were included in this meta-analysis if the met the following criteria: 1) case-control studies of the correlation between the -173 G > A variant of *MIF* gene and tuberculosis; 2) studies enrolled more than 30 patients; 3) studies provided the genotype frequencies of *MIF* -173 G > A polymorphism in both cases and controls. Figure 1(Fig. 1) summarized the process of identifying eligible studies.

### Data extraction

Extraction of the data was done by two independently authors. The following data were collected from each study including the first author's name, publication year, ethnicity of participants, genotyping methods of -176 G > C polymorphism, the sample size, and the genotype frequencies of the variant in both cases and controls.

### Statistical analysis

We performed the present meta-analysis using RevMan 5.0 software which was provided by the Cochrane Collaboration (Version 5.3. Copenhagen: The Nordic Cochrane Centre, The Cochrane Collaboration, 2014). All of the data in the studies are dichotomous data expressed as odds ratios (ORs) with 95 % confidence intervals (CIs) to assess the association between *MIF* -137 G > C gene polymorphism and TB. Hardy-Weinberg equilibrium (HWE) for each study was determined by the chi-square test. 

Statistical heterogeneity among the studies was evaluated using the Q-test and I^2^-test. The pooled ORs were calculated using the fixed-effect inverse variance analysis method or random-effect model (when heterogeneity among the studies was observed) for the allelic comparison (C v G) and genotypic comparisons of codominant (CG vs GG and CC vs GG), dominant (CG +CC vs GG), and recessive (CC vs CG+GG) genetic inheritance models. The significance of the pooled OR was assessed by the Z-test, and *P* < 0.05 was considered to be statistically significant. Publication bias was estimated by funnel plot. 

## Results

After our selection, five case-control studies satisfied the inclusion criteria for our meta-analysis (Gomez et al., 2007[9]; Sadki et al., 2010[26]; Li et al., 2012[15]; Hashemi et al., 2015[12]; Liu et al., 2016[16]). Characteristics of included studies are summarized in Table 1(Tab. 1). 

**Figure 2:**
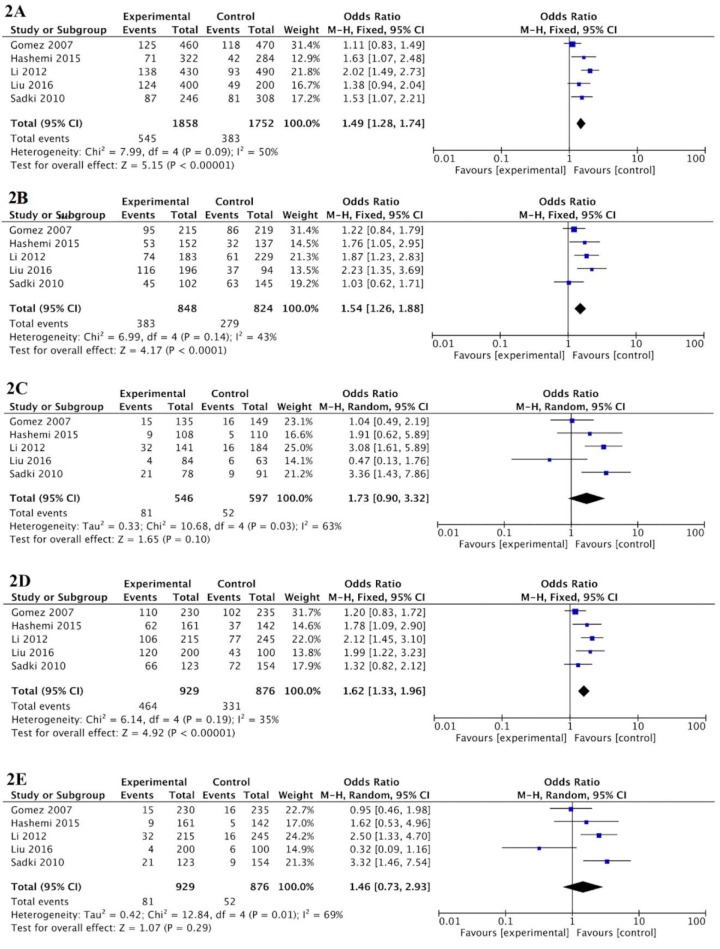
Forest plot of tuberculosis risk associated with *MIF* -173 G > C polymorphism. 2A. Allelic model (C vs G). 2B. Codominant model (CG vs GG). 2C. Codominant model (CC vs GG). 2D. Dominant model (CG+CC vs GG). 2E. Recessive model (CC vs CG+GG). The squares and horizontal lines correspond to the study-specific OR and 95 % CI, respectively. The area of the squares reflects the study-specific weight. The diamond represents the pooled results of OR and 95 % CI.

Five studies involving 929 cases and 876 controls were pooled together for assessment of the overall association between *MIF* -173 G > C variant and the risk of tuberculosis. As shown in Figure 2(Fig. 2), the finding proposed that the *MIF* -173 C allele significantly increased the risk of PTB (OR = 1.49, 95 %CI = 1.28-1.74, p < 0.00001). Furthermore, the *MIF* -173 G > C variant increased the risk of TB in codominant (OR = 1.54, 95 %CI = 1.26-1.88, p < 0.0001; CG vs GG), and dominant (OR = 1.62, 95 %CI = 1.33-1.96, p < 0.00001; GC+CC vs GG) inheritance models tested. But the variant was not associated with TB in recessive inheritance model (OR = 1.46, 95 %CI = 0.73-2.93, p = 0.290; CC vs GC+GG). 

**Figure 3:**
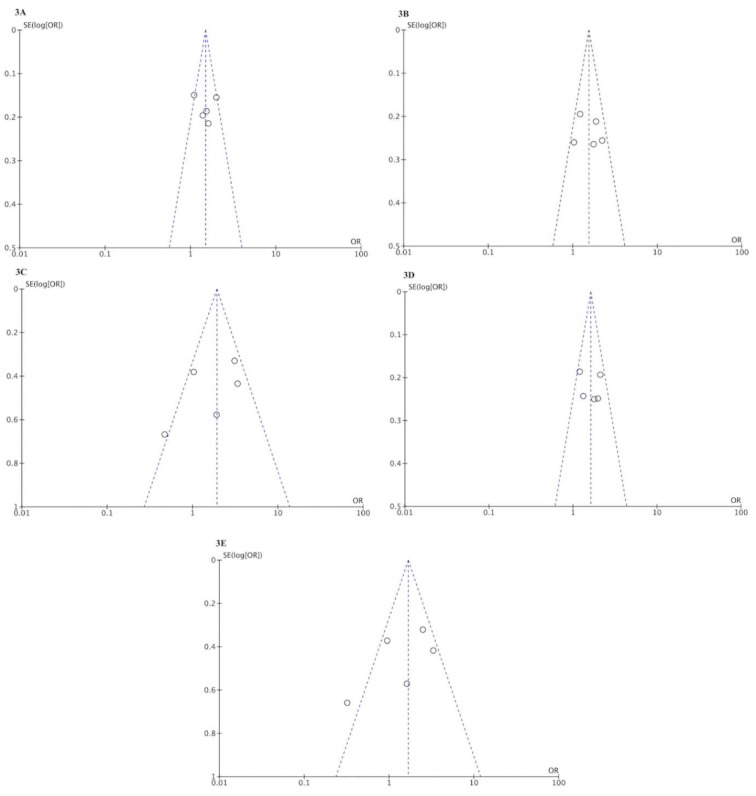
Funnel plots in the meta-analysis of the association between *MIF* -173 G > C polymorphism and tuberculosis risk. 3A. Allelic model (C vs G). 3B. Codominant model (CG vs GG). 3C. Codominant model (CC vs GG). 3D. Dominant model (CG+CC vs GG). 3E. Recessive model (CC vs CG+GG).

A funnel plot was generated as a visual aid to detect risk of publication bias (Figure 3(Fig. 3)).

## Discussion

In the current meta-analysis, we summarized all of the available data regarding the association between *MIF* -173 G > C variant and TB risk. Our findings indicated that *MIF* -173 G > C polymorphism significantly increased the risk of TB susceptibility in codominant, dominant and allelic models. 

MIF plays important functions in regulation of inflammation and innate immune response. Interestingly, MIF signaling pathway counteracts glucocorticoids regulatory effects in immune system. (Xu et al., 2013[31]). Several studies proposed that *MIF* polymorphism increased the risk of immune disease. Liu et al. (2014[17]) revealed that *MIF* rs755622 polymorphism may be a risk factor for new-onset Graves' disease in a Taiwanese Chinese population. 

A meta-analysis performed by Hao et al (Hao et al., 2013[10]) showed that *MIF* -173 G > C polymorphism contributed to the susceptibility of inflammatory bowel disease (IBD). In contrast, the findings of a meta-analysis performed by Falvey et al. (2013[8]) did not support an association between *MIF* -173 G > C polymorphism and susceptibility to IBD in Caucasian subjects.

Zhang et al. (2015[34]) carried out a meta-analysis to find out the impact of *MIF* -173 G > C polymorphism and cancer risk. They found that this variant was significant associated with cancer risk. The findings of another meta-analysis proposed that *MIF* -173 G/C gene polymorphism would be a risk factor for the gastrointestinal cancer and hematological malignancy (Tong et al., 2015[30]).

It has been shown that *MIF* -173 G > C gene polymorphism may be associated with renal disease susceptibility, particularly in children. Moreover, the findings of a meta-analysis designated that this variant may be related to glucocorticoid resistance in child patients with idiopathic nephrotic syndrome (Tong et al., 2015[29]). A meta-analysis performed by (Kaalla et al., 2013[13]) showed that *MIF* -173 G > C variant was not associated with juvenile idiopathic arthritis.

There are several limitations of the current meta-analysis. First, only published data were entered in a few databases, consequently a publication bias may have happened. Second, the predisposition to TB is complex and in most cases does not depend on a single gene polymorphism, but somewhat on many gene variants or gene-environment interaction. 

In conclusion, the findings of this meta-analysis support an association between *MIF* -173 G > C variant and risk of TB.

## Conflict of interest

The authors declare no conflicts of interest.

## Figures and Tables

**Table 1:**
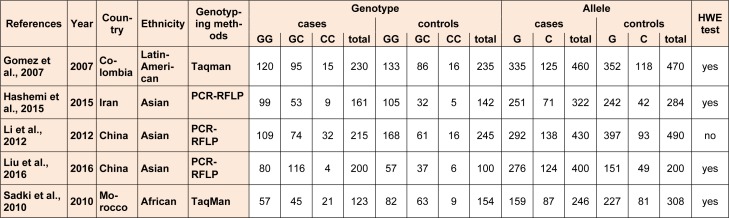
Baseline characteristics of all included study
